# Supplementary material for: Ohmic transition at contacts key to maximizing fill factor and performance of organic solar cells
Source: Nat Commun. 2018 Aug 15;9:3269. doi: 10.1038/s41467-018-05200-w (PMC6093871; doi:10.1038/s41467-018-05200-w)
Supplement: Supplementary file 1 — Supplementary Information [file 41467_2018_5200_MOESM1_ESM.pdf]

Ohmic transition at contacts key to maximizing fill factor and performance of organic solar cells

Jun-Kai Tan,[1,2] Rui-Qi Png,[1,2] Chao Zhao[1,2] and Peter K.H. Ho[1,2]*

[1] Department of Physics, National University of Singapore, Lower Kent Ridge Road, S117550, Singapore
[2] Solar Energy Research Institute of Singapore, National University of Singapore, Engineering Drive 1, S117574, Singapore

* Correspondence to: P.K.H.H. (phyhop@nus.edu.sg)

Supplementary Information

Contents

Supplementary Figure 1 2

Supplementary Table 1 3

Supplementary Note 1: Fermi-Level Pinning and Effective Work Function 4

Supplementary Note 2: Fermi-Level Pinning Calculations 5

Supplementary References 7

Supplementary Figure 1

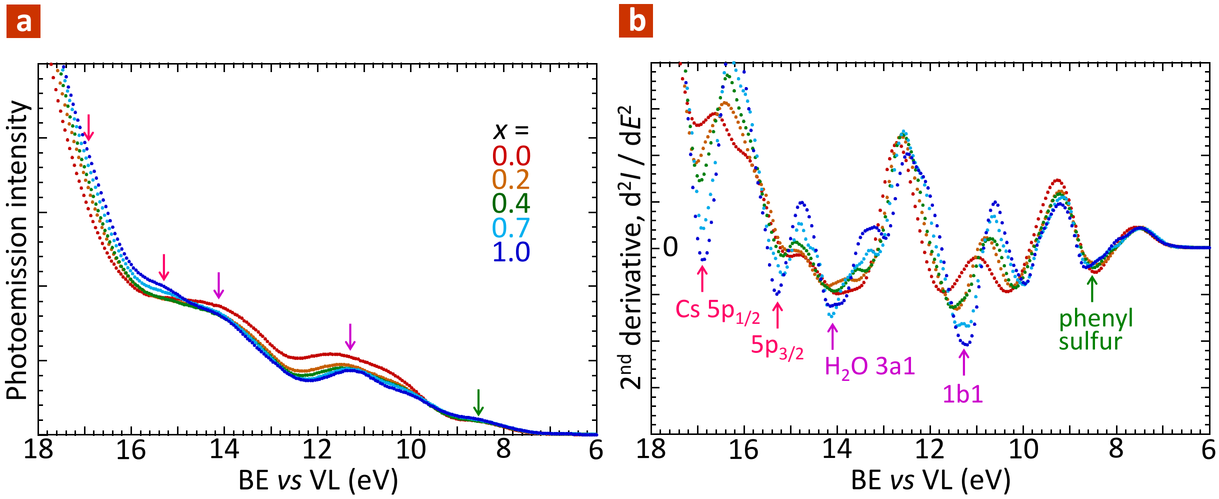

Supplementary Figure 1. Valence band spectrum of PEDT: PSSCs$_x$H$_{1-x}$ films. (a) Valence band spectrum, plotted against binding energy (BE) relative to vacuum level (VL) for film Arrows indicate peak positions determined from second-derivative spectrum. **(b)** Second-derivative spectrum, obtained by digital derivatization of the spectrum in (a) with appropriate smoothing. Spectral assignments are marked. Phenyl refers to the localized phenyl MO of PSS; sulfur refers to the localized (lone-pair) sulfur MO of PEDT. Molecular H_2O is bound to the $-SO_3^-$…Cs^+ ion clusters, but not $-SO_3H$. The constant energies of these features reveal the absence of any contribution from a varying surface-dipole layer to the work function shift. Assignments are based on Chia et al[1] for phenyl and sulfur and Winter et al for the others.[2]

Supplementary Table 1

Parameter		ϕ (eV)					
		**5.17**	**5.10**	**5.03**	**4.94**	**4.88**	**4.72**
Electron mobility	μ_n	9 x 10^{-4} cm^2 V^{-1} s^{-2}					
Hole mobility	μ_p	1 x 10^{-4} cm^2 V^{-1} s^{-2}					
Electron density, at electron contact	N_n	2.0 x 10^{17} cm^{-3}					
Hole density, at hole contact	P_p	2.0	1.7	1.4	0.9	0.6	0.1
		x 10^{17} cm^{-3}					
Recombination factor	ξ	0.2					
Zero-K built-in potential	V_o	0.73 V					
Dielectric constant	ε_r	2.0					

Supplementary Table 1. Device parameter set employed for simulation of bulk *JV* characteristics for PEDT: $PSSCs_xH_{1-x}$/ P3HT: PCBM/ Ca solar cells. The parameters have been validated in crosslinked donor polymer network: fullerene solar cells with well controlled donor−acceptor morphology over wide thickness range.[3] P_p is a variable scaled by ($\phi - \phi_{pin}$).

Supplementary Note 1: Fermi-Level Pinning and Effective Work Function

We use ϕ to denote the vacuum work function of the electrode, in this case PEDT: PSS(Cs_xH_{1-x}), which is the energy difference between its Fermi level (FL) and the outside vacuum level (VL). This can be measured, for example by ultraviolet photoemission spectroscopy (UPS).[4-6] We denote the effective work function of the electrode in contact with the semiconductor by ϕ_{eff}, which is the energy difference between FL of the electrode and the effective VL of the bulk of the semiconductor.[7] The effective VL is what pertains when the semiconductor is notionally split to create a vacuum interface but without creating a dipole layer at the vacuum interface. ϕ_{eff} rather than ϕ is the quantity that is relevant to energy-level alignment in devices. It can be inferred from V_{bi} measurements in diodes, assuming ϕ_{eff} at one of the contacts is known *a priori*, for example through systematic correlation.[7] Surveys of polymer organic semiconductor diodes suggest that ϕ_{eff} is a well-defined and portable under some conditions, in particular in the absence of Fermi-level (FL) pinning.[7] When ϕ exceeds the work function for the onset of FL pinning ϕ_{pin}, charge transfer to the semiconductor band edge occurs, which opens a VL offset that greatly slows down the creep of FL up the density-of-states of the semiconductor.[8,9] As a consequence ϕ_{eff} appears to be pinned at ϕ_{pin}. The location of ϕ_{pin} can thus be obtained from the interpolated knee in the plot of ϕ_{eff} against ϕ. The resultant accumulation of carrier density in an ultrathin layer with diffused tail at the semiconductor side of the contact, which we refer to variously as the 'δ-doped layer' or 'δ-density', can be measured and quantified by subgap electroabsorption spectroscopy.[10]

Supplementary Note 2: Fermi-Level Pinning Calculations

Calculations were performed at 290 K with the following parameters:

Parameter	Symbol	Value
Gaussian density-of-states	σ_G	3 x 10^{13} cm^{-2}
Static dielectric constant	ε_r	2.0[a]
Effective dielectric thickness	d	2.5 nm
Contact double-layer capacitance	C_{dl}	0.7 μF cm^{-2}

Footnotes:

[a] We used a ε_r lower than 3 because of the absence of the conjugated polymer in the local ion cluster.

In a typical calculation, a certain density-of-states (DOS) function for the semiconductor is assumed. Then for each assumed effective work function of the hole contact ϕ_{eff} given by the work function for the semiconductor, we computed the vacuum-level offset at the contact $\Delta\phi_{dl}$ and the associated work function of the electrode ϕ, according to: $\phi = \phi_{eff} + \Delta\phi_{dl}$. $\Delta\phi_{dl}$ is given by the voltage drop associated with the double-layer capacitance due to the accumulated hole density and its image, which in the zero-K limit is given by: $\Delta\phi_{dl} = \frac{e\sigma}{C_{dl}}$, where σ is the accumulated hole density given by $\sigma = \int_{\varepsilon_F}^{\infty} N_i(\varepsilon_i)(1 - f_i(\varepsilon_i))d\varepsilon_i$, where $N_i(\varepsilon_i)$ is the DOS and $f_i(\varepsilon_i)$ is the Fermi-Dirac function, and C_{dl} is the double-layer capacitance given by $C_{dl} = \frac{\varepsilon_o \varepsilon_r}{d}$, where ε_o is the vacuum permittivity, ε_r is the local dielectric constant, and d is the effective double-layer thickness. For an interface with a perfect conductor, d is given by half the distance to the image charge. We have checked that a variation in $\Delta\phi_{dl}$ by up to a factor of three, due to uncertainty in C_{dl}, does not change the form of the plots. The FL pinning onset work function ϕ_{pin} is established from the kink in the ϕ_{eff} *vs* ϕ plot. This was computationally obtained by intersecting the unity-slope

straight line in the $\phi < \phi_{pin}$ regime, with a straight line fitted over $\phi_{pin} + 0.1 < \phi < \phi_{pin} + 0.4$ eV regime, following the usual experimental practice.

For a DOS with a hemi-gaussian frontier region with integrated hemi-gaussian DOS of 4 x 10^{13} cm^{-2}, our simulation gives $\phi_{pin} \approx (2.35 \pm 0.02) * \sigma_G$, where σ_G is the gaussian (standard deviation) width. For comparison, the ionization energy I_E conventionally defined by extrapolated onset of the highest-occupied molecular orbital (HOMO) band edge is at 2.00 $*\sigma_G$. Therefore in the absence of polarization-induced band-bending, Fermi-level pinning is expected to occur very close to the conventionally-defined band edge, at *ca*. 0.35 $*\sigma_G$ into the gap from the band edge. This amounts to 63 meV for a typical σ_G of 0.18 eV. Furthermore, we found for σ_G of 0.18 eV that contact resistivity scales as: $\rho_c \sim \sigma^{-0.82}$ for ϕ in the range of $\phi_{pin} \pm 0.2$ eV.

Supplementary References

1. Chia, P. J. *et al.* Direct evidence for the role of the Madelung potential in determining the work function of doped organic semiconductors. *Phys. Rev. Lett.* **102**, 096602 (2009).
2. Winter, B. *et al.* Electron binding energies of aqueous alkali and halide ions: euv photoelectron spectroscopy of liquid solutions and combined ab initio and molecular dynamics calculations. *J. Am. Chem. Soc.* **127**, 7203 (2005).
3. Liu, B., Png, R. Q., Tan, J. K. & Ho, P. K. H. Evaluation of built-in potential and loss mechanisms at contacts in organic solar cells: device model parameterization, validation, and prediction. *Adv. Energy Mater.* **4**, 1200972 (2014).
4. Koch, N. Organic electronic devices and their functional interfaces. *ChemPhysChem* **8**, 1438 (2007).
5. Braun, S., Salaneck, W. R. & Fahlman, M. Energy-level alignment at organic/metal and organic/organic interfaces. *Adv. Mater.* **21**, 1450 (2009).
6. Hwang, J., Wan, A. & Kahn, A. Energetics of metal–organic interfaces: new experiments and assessment of the field. *Mater. Sci. Eng. R-Rep.* **64**, 1 (2009).
7. Zhou, M. *et al.* Effective work functions for the evaporated metal/organic semiconductor contacts from in-situ diode flatband potential measurements. *Appl. Phys. Lett.* **101**, 013501 (2012).
8. Ishii, H., Sugiyama, K., Ito, E. & Seki, K. Energy level alignment and interfacial electronic structures at organic/ metal and organic/ organic interfaces. *Adv. Mater.* **11**, 605 (1999).
9. Tengstedt, C. *et al.* Fermi-level pinning at conjugated polymer interfaces. *Appl. Phys. Lett.* **88**, 053502 (2006).
10. Zhou, M. *et al.* The role of delta-doped interfaces for Ohmic contacts to organic semiconductors. *Phys. Rev. Lett.* **103**, 036601 (2009).
